# The importance of the altricial – precocial spectrum for social complexity in mammals and birds – a review

**DOI:** 10.1186/s12983-016-0185-6

**Published:** 2017-01-18

**Authors:** Isabella B. R. Scheiber, Brigitte M. Weiß, Sjouke A. Kingma, Jan Komdeur

**Affiliations:** 1 The University of Groningen, Behavioural and Physiological Ecology, Groningen Institute for Evolutionary Life Sciences (GELIFES), Nijenborgh 7, 9747 AG Groningen, The Netherlands; 2 Behavioural Ecology Research Group, University of Leipzig, Faculty of Bioscience, Pharmacy and Psychology, Institute of Biology, Talstraße 33, 04103 Leipzig, Germany; 3 Department of Primatology, Max Planck Institute for Evolutionary Anthropology, Deutscher Platz 6, 04103 Leipzig, Germany

**Keywords:** Altricial-precocial spectrum, Birds, Mammals, Social behaviour, Social cognition

## Abstract

Various types of long-term stable relationships that individuals uphold, including cooperation and competition between group members, define social complexity in vertebrates. Numerous life history, physiological and cognitive traits have been shown to affect, or to be affected by, such social relationships. As such, differences in developmental modes, *i.e.* the ‘altricial-precocial’ spectrum, may play an important role in understanding the interspecific variation in occurrence of social interactions, but to what extent this is the case is unclear because the role of the developmental mode has not been studied directly in across-species studies of sociality. In other words, although there are studies on the effects of developmental mode on brain size, on the effects of brain size on cognition, and on the effects of cognition on social complexity, there are no studies directly investigating the link between developmental mode and social complexity. This is surprising because developmental differences play a significant role in the evolution of, for example, brain size, which is in turn considered an essential building block with respect to social complexity. Here, we compiled an overview of studies on various aspects of the complexity of social systems in altricial and precocial mammals and birds. Although systematic studies are scarce and do not allow for a quantitative comparison, we show that several forms of social relationships and cognitive abilities occur in species along the entire developmental spectrum. Based on the existing evidence it seems that differences in developmental modes play a minor role in whether or not individuals or species are able to meet the cognitive capabilities and requirements for maintaining complex social relationships. Given the scarcity of comparative studies and potential subtle differences, however, we suggest that future studies should consider developmental differences to determine whether our finding is general or whether some of the vast variation in social complexity across species can be explained by developmental mode. This would allow a more detailed assessment of the relative importance of developmental mode in the evolution of vertebrate social systems.

## Background

Studies that investigate vertebrate social life from various perspectives (*i.e.* behavioural, neurobiological, physiological and cognitive components) are on the leading edge of scientific investigations both from an evolutionary and mechanistic point of view (*e.g.* [1–7]). The general characteristic that defines complex social systems in vertebrates is that animals live in long-term stable groups of multiple generations, which allows for repeated interactions with differently familiar individuals. These interactions encompass various forms of cooperation and competition over resources, and require considerable learning over the course of development [8]. As such, various factors, including life history, physiology and brain structure, which may be associated with potential differences in cognitive abilities, shape individuals’ engagement in complex social interactions.

**Fig. 1:**
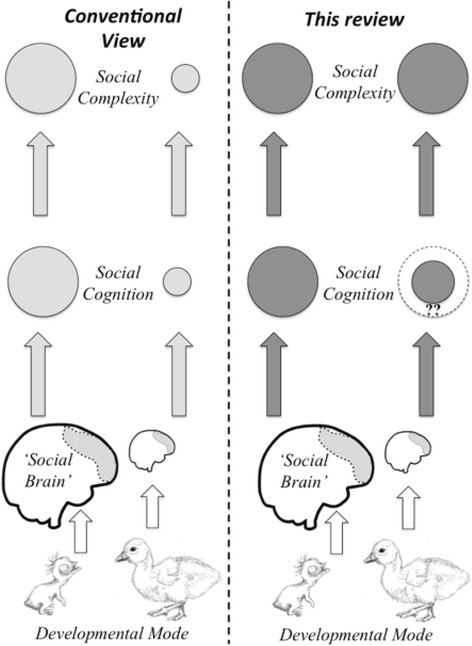
Schematic representation of the relationship between developmental mode [altricial offspring left, precocial offspring right], social brain size, social cognition and social complexity. Whereas the influence of developmental mode on variation in the ‘social brain size’ and ensuing cognitive abilities and the deduced effects on social complexity are well established (*conventional view*, light grey pathway; (*e.g*. [4, 6, 9–12, 14, 15, 17]), we emphasize a different idea in this review, namely that social complexity may not be associated with developmental mode despite differences in brain size (dark grey pathway; see Table 1). Whether socio-cognitive skills are similar or reduced in precocial and altricial species, however, cannot be determined due to the lack of systematic studies addressing these questions (Displayed by ‘??’ as well as a dashed circle of social cognition in the right pathway)

One often-neglected feature that may underlie variation in the complexity of social systems is a differentiation of species with respect to their developmental mode*, i.e*. the ‘altricial-precocial’ spectrum. Based on inferences from indirect factors such as life history and brain size, several authors have recently hinted at a connection between developmental modes, brain size and variation in the complexity of social life, bonding systems and cognition (*e.g*. [4, 6, 7, 9–17]). From a mechanistic point of view, such a pathway from developmental mode to social complexity seems plausible (see Fig. 1, “conventional view”), but the explicit relationship between developmental mode and social complexity has received limited attention. Accordingly, we do not know if evolutionary history of social complexity supports this link nor, if it exists, the causality between developmental mode and social complexity. One of our aims here is to survey the existing literature to determine whether social complexity is related to variation in developmental mode in mammals and birds, the two most extensively studied vertebrate taxa in this regard.

The alterations in brain size with an alleged impact on cognitive abilities in species along the altricial-precocial spectrum have led to the prevalent notion that larger-brained species also have a more complex social life (*e.g*. [4–6, 18]). Alternatively, there is recent debate on whether complex social life – indeed - requires large brains and highly complex cognitive skills or whether similarly complex sociality can be attained through variation in brain composition (*i.e.* ‘cerebrotypes, see below) and/ or simpler cognitive mechanisms (*e.g*. [1, 7, 19–21]). This dichotomy in thinking requires a thorough assessment, which we provide in this review. Our expectation is that complex social systems can similarly be found in birds and mammals regardless of their developmental mode as complex social behaviour is found throughout the entire animal kingdom. Therefore, we will evaluate, whether social behaviours are expressed similarly or differently in precocial and altricial species. We aim to assess whether the inferred indirect link of a relationship between developmental mode and social complexity via variation in relative brain size is supported or if there is a direct link between developmental mode and social complexity independent of brain size variations (Fig. 1). In this context, we will focus on similarities and differences of the ‘social brain’, as it is now clear that the brain circuits which regulate social behaviour in non-mammalian vertebrates are homologous to those found in mammals [22–25]. We will also summarize the ongoing debate about whether coping in a social world requires high-level cognition [1, 7, 16, 21] and how variation in developmental modes affects cognitive abilities.

### The altricial precocial spectrum in mammals and birds

The altricial-precocial spectrum describes the degree of behavioural and morphological maturation of offspring at the moment of birth or hatching [26]. In precocial species, young require limited parental care and are relatively mature, mobile and can either mainly feed self-sufficiently (precocial birds) or forage independently from early on while still being nursed (precocial mammals). Altricial young, in contrast, are initially incapable of moving around on their own and require extensive parental care, like brooding or food provisioning. The most extreme developmental modes are super-precociality, where offspring are completely independent immediately after hatching or birth (as in *e.g.* megapodes, black-headed duck or wildebeest [27–29]), or super-altriciality, where offspring hatch or are born more or less naked with their eyes closed (as in *e.g.* cricetid rodents, canids [30], monotremes [31] and marsupials [14, 32, 33], passerines or parrots [for review [34]). A recent re-evaluation of the altricial-precocial classification of species by Ligon & Burt [35] denominated 8890 species out of the 9993 extant species of birds to have altricial development [36]. The distribution of developmental modes in the ± 5420 mammal species is not as straightforward [30], but seems to be correlated with body size or mass, gestation period, and/or number of offspring: larger mammals are more likely to produce very few precocial young per litter [30, 37–40] whereas small mammals are more likely altricial and produce more young. One notable exception, amongst others, are bats (Chiroptera), which presumably produce small altricial litters due to adaptation for flight [41]. Starck & Ricklefs [26] provide a detailed summary on the evolutionary diversification of life histories in relation to the marked variation in development mode, parental care and rate of growth in primarily birds, with a short section devoted to mammals. It is now well established that these different developmental trajectories have long-term consequences in various aspects of endocrine, reproductive or other physiological mechanisms. In this review, we will, therefore, focus on another feature, *i.e*. the influence of developmental modes on the complexity of social systems and its underlying mechanisms only. We focus on several important social and cognitive features (see Table 1; detailed below) that we deem essential for complex sociality, to determine if these can be found in avian and mammalian species along the altricial-precocial spectrum. As there is only a very limited number of studies available that specifically incorporate the developmental mode in questions pertaining to complex sociality, and because social complexity is difficult to comparably quantify (but see [42] for a recent review and new definition), we were unable to perform a rigid meta-analysis. Specifically, we first summarise the possible features that we assume reflect social complexity. Second, we describe the cognitive features that are considered to be necessary in order to establish, maintain and manage complex social relationships. Finally, we compiled a thorough collection of studies connecting developmental mode with 15 different features of social complexity, including social (*e.g.* affiliative behaviour or long-term bonds) and cognitive (*e.g.* kin recognition) features of altricial and precocial mammals and birds (see Tables 1 and 2 for definitions of the features used in this review).

**Table 1:** Various social (top) and cognitive (bottom) features of mammals (M) and birds (B) with respect to their developmental mode (A = altricial, P = precocial)

*Characteristics of social complexity*	Subcategory	Taxonomic Class	Developmental Mode	Examples	Reference
*Social features*					
Long-term, extended bonds/Valuable relationships	Kin	M	A	Primates - Review; Yellow baboon (*Papio cynocephalus);* Mountain gorilla (*Gorilla beringei beringei*); Gelada (*Theropithecus gelada*); Raccoon (*Procyon lotor*)	[71, 89, 211–213]
		M	P	Sperm whale (*Physeter macrocephalus*); Cetaceans – Review; African elephant (*Loxodonta africana*); Wild boar (*Sus scrofa*); Horse (*Equus cabalus)*	[214–218]
		B	A	Raven (*Corvus corax);* Jackdaw *(C. monedula;);* Rook *(C. frugilegus*);	[6, 72, 113, 219–221]
		B	P	Greylag goose (*Anser anser*); Barnacle goose (*Branta leucopsis*)	[86, 222, 223]
	Unrelated individuals	M	A	Bechstein bat (*Myotis bechsteinii*); Chimpanzee (*Pan troglodytes*)	[130, 224, 225]
		M	P	Horse*;* wild Giraffe (*Giraffa camelopardalis*)	[226, 227]
		B	A	various species – Review; Long-tailed manakin (*Chiroxiphia linearis*); Laysan albatross (*Phoebastria immutabilis*)	[228–231]
		B	P	various species - Review	[231–233]
Affiliative behaviours	Allogrooming/Allopreening	M	A	Chimpanzee; Rhesus macaque (*Macaca mulatta*); Vervet monkey (*Chlorocebus pygerythrus*) Columbian ground squirrel (*Spermophilus columbianus)*	[225, 234–237]
		M	P	Horse; Cow (*Bos taurus*)	[114, 238, 239]
		B	A	Green woodhoopoe (*Phoeniculus purpureus*); various Corvid specs. (raven, jackdaw, rook)	[6, 106, 240]
		B	(semi-) P	Common guillemot (*Uria aalge*)	[241]
	Allofeeding/Food sharing	M	A	various species - Review	[242]
		M	P	various species (Cetaceans) - Review	[242]
		B	A	Jackdaw; Eurasian siskin (*Carduelis spinus*); Cliff swallows, (*Hirundo pyrrhonota*); ± all cooperative breeders, *e.g.* Arabian babbler (*Turdoides squamiceps*)	[243–247]
		B	P	Barnacle goose; Greylag goose	[223, 248, 249]
	Behavioural synchrony	M	A	Primates - Review	[17]
		M	P	Sperm whales (*Physeter macrocephalus*); Indian Ocean bottlenose dolphins (*Tursiops aduncus*)	[250, 251]
		B	A	Jackdaw; Cockatiel (*Nymphicus hollandicus*)	[6, 252]
		B	P	Red junglefowl (*Gallus gallus);* Greylag goose	[253, 254]
	Spatial (close) proximity	M	A	various primates and non-primates – Review; Tasmanian devil (S*arcophilus harrisii);* Collared peccary (*Pecari tajacu*).	[92, 255, 256]
		M	P	African elephant; feral goat (*Capra hircus*); Cow;	[87, 93, 257]
		B	A	various Corvid spp. (raven, jackdaw, rook, New Caledonian crow (*C. moneduloides*))	[6, 83, 93]
		B	P	Barrow’s goldeneye (*Bucephala islandica*); Greylag goose	[86, 88]
Coalitions/Alliances		M	A	Spotted hyenas (*Crocuta crocuta*), various primates and non-primates – Review, Vervet monkey	[101, 236, 258, 259]
		M	P	Indian Ocean bottlenose dolphins; Various ungulates - Review	[251, 258]
		B	A	various Corvid spp. (raven, jackdaw, rook, carrion crow (*Corvus corone*))	[83, 100, 258, 260]
		B	P	Greylag goose; Bewick’s swan (*Cygnus bewickii*); Eider duck (*Somateria mollissima*)	[258, 261–264]
Communal defence		M	A	Crested black macaque (*Macaca nigra*)	[103]
		M	P	Chamois (*Rupicapra rupicapra*);	[102]
		B	A	Montagu’s harrier (*Circus pygargus*); Sabine’s gull (*Xema sabini*)	[265, 266]
		B	P	White-fronted goose (*Anser albifrons*)	[267]
Communal/Cooperative breeding		M	A	various species - Review	[107, 125, 268]
		M	P	various species – Review; *e.g.* Degu (*Octogon degus*); African striped mouse (*Rhabdomys pumilio*)	[105, 107, 110, 125, 268]
		B	A	various species - Review	[104, 109, 269]
		B	P	various species – Review; White-winged trumpeter (*Psophia leucoptera*); Buff-throated partridge *(Tetraophasis szechenyii);* Black-breasted wood-quail (*Odontophorus leucolaemus*); Common moorhen (*Gallinula chloropus*); Dusky moorhen (*G. tenebrosa);* pukeko (*Porphyrio melanotus*)	[104, 109, 120–123, 269–271]
Conflict resolution (*e.g.* reconciliation/consolation; redirected aggression)		M	A	various Primates – Review; Wolf (*Canis lupus*); Spotted hyena; Meerkat (*Suricata suricatta*)	[74, 111, 115, 272–275]
		M	P	Bottlenose dolphin (*Tursiops truncatus*); Horse;	[114, 276, 277]
		B	A	various Corvid specs. (raven, rook)	[112, 113, 278]
		B	P	Greylag goose	[261]
Social support/Social buffering		M	A	various species – Review; Barbary macaques (*Macaca sylvanus*); Wistar rat (*Rattus norvegicus domesticus*); Domestic pig (*Sus scrofa domestica*)	[116, 279–281]
		M	P	Guinea pig (*Cavia aperea* and *Galea monasteriensis*)	[282]
		B	A	various Corvid spp. (raven, jackdaw, rook)	[6, 240]
		B	P	Domestic chicken (*Gallus gallus domesticus*); Greylag goose - Review	[117, 283]
*Cognitive features*					
Recognition of close kin	Parent-offspring	M	A	Seba’s short-tailed bat (*Carollia perspicillata*); Brandt’s vole, (*Lasiopodomys brandti)*	[160, 284]
		M	P	Australian sea lion (*Neophoca cinerea*); Goat	[159, 162]
		B	A	Cliff swallow (*Petrochelidon pyrrhonota);* Cave swallows (*P. fulva*) Black-legged kittiwake (*Rissa tridactyla);* European storm petrel (*Hydrobates pelagicus*); Spectacled parrotlet (*Forpus conspicillatus*) *pyrrhonota*); Black redstart (*Phoenicurus ochruros*)	[157, 158, 161, 170, 285, 286]
		B	P	Black swan (*Cygnus atratus*)	[287]
	Offspring-parent	M	A	Common racoon (*Procyon lotor*)	[288]
		M	P	Fallow deer (*Dama dama*); Red deer (*Cervus elaphus*); Sheep (*Ovis aries*)	[165, 175, 289]
		B	A	Bell miner (*Manorina melanophrys*)	[290]
		B	P	Saunder’s gull (*Saundersilarus saundersi*); Greylag goose	[163, 164]
	Sibling	M	A	Spotted hyena; House mouse (*Mus musculus domesticus*)	[171, 291]
		M	P	Spiny mouse, (*Acomys cahirimus*); Beaver (*Castor canadensis*)	(reviewed in [168] Table 4, [292])
		B	A	various species – Review; Spectacled parrotlet; Barn owl (*Tyto alba*); Barn swallow (*Hirundo rustica*); Long-tailed tit (*Aegithalos caudatus*)	[154, 167–169, 176, 293]
		B	P	Greylag goose	[166]
Recognition of distant kin		M	A	Belding’s ground squirrel (*Spermophilus beldingi*); White-footed mouse (*Peromyscus leucopus*); Oldfield mouse (*P. polionotus rhoadsi*); Rat	[294]
		M	P	Spiny mouse	[294, 295]
		B	A	Zebra finch (*Taeniopygia guttata*)	[296]
		B	P	Japanese quail (*Coturnix japonica*)	[156]
Recognition of unfamiliar kin		M	A	House mouse; Meerkat; Belding’s ground squirrel; White-footed mouse; Rat	[171, 177, 294]
		M	P	Iberian red deer (*Cervus elaphus hispanicus*)	[297]
		B	A	Zebra finch; Japanese quail; Siberian jay (*Perisoreus infaustus*)	[155, 156, 298]
		B	P	Peacock (*Pavo cristatus*); wild Turkey (*Meleagris gallopavo*)	[178, 299]
Individual recognition		M	A	Dwarf mongoose, (*Helogale parvula*)	(recent review [142, 300])
		M	P	domestic goat; African elephant; Horse	[159, 301, 302]
		B	A	Barn owl; Zebra finch Black redstart (*Phoenicurus ochruros*)	[158, 176, 303]
		B	P	Greylag goose	[166]
Long-term memory		M	A	Guinea baboon (*Papio papio*); Cotton-top tamarin (*Saguinus oedipus*)	[179, 181]
		M	P	Goat; Northern fur seal; (*Callorhinus ursinus*); Australian sea lion; Horse	[180, 183–185]
		B	A	various Corvid specs. (raven, jackdaw, rook, Jungle crow (*Corvus macrochynchos*); Pigeon (*Columba livia*)	[6, 179, 182, 186]
		B	P	Greylag goose	[187]
Keeping track and deducing unknown relationships (transitive inference)		M	A	Rhesus macaque; Black lemur (*Eulemur macaco)*, Common brown lemur (*E. fulvus*); House mouse	[304–306]
		M	P	Horse	[185]
		B	A	various Corvid specs. (Pinyon jay (*Gymnorhinus cyanocephalus*), Clark’s nutcracker (*Nucifraga columbiana*), Azure-winged magpie (*Cyanopica cyanus*), Western scrub jay (*Aphelocoma californica*)); Pigeon	[193, 307–309]
		B	P	Chicken, Greylag goose	[310–312]
3^rd^ party recognition		M	A	Primates – Review; Chimpanzee; Spotted hyena; Meerkat: domestic Dog (*Canis lupus familiaris*)	[140, 258, 259, 273, 313–315]
		M	P	Fallow deer; Przewalski horse (*Equus ferus przewalskii*)	[316, 317]
		B	A	various Corvid specs. (raven, rook)	[112, 278]
		B	P	Greylag goose	[261, 318]
Social learning		M	A	Meerkat	[59, 202, 319]
		M	P	African elephant; Thornicoft’s giraffe (*Giraffa camelopardalis thornicrofti*); various Cetaceans – Review; Domestic pig	[196–198, 320, 321]
		B	A	various species – Review; Pigeon; King penguin (*Aptenodytes patagonicus*)	[199–201]
		B	P	Greylag goose	[203]

For a definition of characteristics of social complexity, see Table 2 in the main text. Some features are further classified in significant subcategories

No human studies are included

**Table 2:** Glossary definition of characteristics of social complexity (social and cognitive features; see also Table 1)

Characteristics of social complexity	Definition
Social features	
Long-term, extended family bonds	Family relationships, which last beyond independence of offspring, including multi-generational family units
Valuable relationships	Unique history of interactions between two individuals, which leads to a broad variation in the quality of social relationships between individuals within groups rendering some individuals more ‘*valuable*’ than others for each individual in the group. Valuable relationships are characterised by: • Individuals in close proximity • High rates of affiliative behaviours (see below) • Low rates of aggression • Social support (see below)
Affiliative behaviours	Behaviours, which promote socio-positive relationships between two individuals or group cohesion, *e.g*. grooming
Coalitions/ Alliances	Individuals that jointly participate in aggressive acts against conspecifics or to gain access to resources form transitory (short-term) *coalitions* or long-term *alliances*
Communal Defence	Prey groups actively defend themselves or their offspring by attacking or mobbing a predator, rather than allowing themselves to be passive victims of predation
Communal/ Cooperative Breeding	Cooperative breeding is a social system, characterised by allo-parental care when more than two individuals of the same species provide care in rearing young. Although sometimes used interchangeably, communal breeding is now often applied to cases in which individuals also share reproduction, *i.e*. when two or more females lay eggs into or rear young within a single nest
Conflict Resolution (Reconciliation, consolation, redirected aggression)	Post-conflict affiliative interactions between former opponents (reconciliation), re-affirmative contacts between the victim of aggression and a bystander (consolation) or an aggressive act by the victim against an uninvolved individual (redirected aggression)
Social support/ social buffering	The stress-reducing effect gained by the presence of (a) social allies (ally)
Cognitive features	
Individual recognition (IR)	The ability to distinguish between different individuals either through recognition of actual individually distinctive features (true IR) or class-level cues, such as familiarity, location, kinship (untrue IR). Kin recognition is an animal’s ability to distinguish between close kin and non-kin
Long-term memory	Information, longer lastingly stored in the brain, which is retrievable over extended periods of time
Transitive Inference (TI)	TI is a form of deductive reasoning that allows one to derive a relation between items that have not been explicitly compared before. In a general form, TI is the ability to deduce that: If A > B and B > C, then A > C. In order to be transitive, relations need an underlying scale.
3rd party recognition	The ability to recognize tertiary relationships between conspecific group members, which involve interactions and relationships in which the observer is not directly involved.
Social learning	A process in which the behaviour of others and its consequences are observed and one’s own behaviour is modified accordingly.

#### Arguments for and against linking social complexity with developmental mode

There are recent claims that the manner and quality of social relationships depends on the developmental mode [5, 6, 10, 17, 43] due to the link of developmental mode and brain development. In mammals, expansion of the cerebral cortex plays a major role in managing social interactions, whereas in birds and seemingly socially complex marsupials, social interactions are regulated by the homologous enlarged telencephalon [43–46], but with keeping in mind that hardly any information on the social system of marsupials is available. The general pattern in birds is that adults in altricial species have relatively large brains compared to adults of precocial species, whereas at hatching the pattern is reversed [47, 48]. Precocial offspring possess relatively large brains due to the fact that neural growth in precocial species takes place in the egg, while in altricial species it occurs after hatching ([47] for review). Due to their extended post-hatching development, altricial bird species might therefore be more skilled in managing social interactions given their larger brains. On the other hand, relative brain size in mammals does not seem to be correlated with developmental mode *per se* [49], but rather is negatively correlated with litter size in altricial species and a reduction in birth rate in precocial species ([14] for review, [50, 51]). The proposed explanation for this pattern is that precocial mammals develop slower and reach sexual maturation later in life than altricial young [51].

Arguments against a relationship between social complexity, brain size variation and developmental mode stem from studies that measured the size of multiple brain regions in a multivariate context in mammals and birds [15, 52–54]. These so-called ‘cerebrotypes’ are defined by comparing the proportional size of different parts of the brain to total brain size. Developmental mode does not seem to have a strong effect on cerebrotypes, as altricial and precocial species are represented in each avian- [12, 54] and mammalian-specific [52] cerebrotype.

Another aspect that supports the notion of similar social complexity in altricial and precocial species are the underlying neuro-endocrinological and molecular mechanisms, which play a central role in the regulation of maternal and other socio-sexual behaviours. These mechanisms involve a range of neuropeptides (*e.g.* β-endorphin, corticotrophin-releasing factor, oxytocin and arginine-vasopressin as well as the avian homologues mesotocin and arginine-vasotocin) and are highly conserved throughout vertebrates of all developmental modes [30, 55–58]. Oxytocin mediates several forms of affiliative behaviours, including parental care, and grooming [3, 59–64], the formation of a pair-bond [65, 66], as well as the establishment of the exclusive bond between mothers and offspring [67]. Oxytocin is also known for its positive impact on the development of trust and recognition of familiar individuals in rodents [68] and estrildid finches [61]. Likewise, the ‘social behaviour network’- brain regions that control social behaviour - is also very highly conserved across the vertebrates [22, 69] irrespective of developmental mode. Precocial and altricial species thus possess a similar neuro-endocrinological tool kit, which is an essential prerequisite for acquiring similarly complex social behaviour. In the following sections, we will review to what extent these similarities and differences in brain structures and physiology translate into similarities or differences in social complexity and cognition.

### Compilation of data

We collected data for this review searching the *Web of Science* to find publications whose title, abstract or key words included any of the following terms: developmental mode/ altricial/ precocial, social system/ social complexity, mammal, bird. We omitted any studies, in which developmental mode and sociality were not defined in the main text. We double-checked information on every publication that seemed suitable for this review, by searching the web for additional information on the correctness of developmental mode and social system on any species given, and excluded species in which these issues were equivocal. We then searched the remaining publications for terms characterizing either social complexity or cognitive features (see Table 2) and compiled relevant publications in Table 1. Whenever possible, we cited published reviews, which contain a wealth of information on various taxa. Finally, we specifically searched for information about social and cognitive features still missing from the table to fill in any missing table cells. In cases where many studies pertained to one topic, we did not list all studies but listed a diverse array of species showing this specific characteristic. Note therefore that our list of species is not exhaustive.

#### Comparing features of social complexity and elaborate social relationships in precocial and altricial species

In vertebrates, the complexity of social systems is not related to the actual number of individuals per group, but rather to the variety of associations and elaborate interactions that group members engage in [70] or, as Bergman & Beehner [42] recently termed it ‘ the number of differentiated relationships’. It is described best by the maintenance of individualized long-term, mutual, dyadic ‘valuable relationships’ (sensu [71]). Valuable relationships are characterised by close proximity between bonded partners, the provision of social support, low rates of aggression and the occurrence of affiliative behaviours, particularly also after conflicts have occurred [71]. Hence, for a comparative study, a pivotal question to assess social complexity is how to measure the strength and/or quality of bonds between individuals [17, 72–74], as not all measures are comparable or, perhaps, of equal importance across species. Therefore, it is especially important to assess a suite of features that may reflect social complexity to make broad inferences about the role of certain factors in explaining that complexity [42]. For example, certain affiliative tactile behaviours, such as feeding or grooming others, are often used as indicators of close bonds between individuals and are expressed similarly in altricial and precocial mammals [75], but are, in contrast to altricial birds, uncommon or absent in many precocial birds [76]. However, both altricial and precocial species express social bonds in a variety of other ways, including vocal and visual displays ([76–81] for a mammalian review) and chemical [82] cues, increased tolerance and spatial proximity [83–85]. In particular, the spatial association between individuals is often used as a proxy for determining social relationships ([86–88], but see [89]). As such, it is now evident from social network analyses [90, 91] that close proximity indeed is a legitimate measure for close affiliative bonds ([92–95], but see [96]). Nearness between individuals that maintain social bonds is found in species of all developmental modes (Table 1). In sum, both altricial and precocial birds and mammals resort to a large variety of displaying affiliative bonds. The lack of any one of these above indicators of social bonds, however, does not necessarily infer weak and/or low quality affiliative relationships between precocial or altricial mammals or birds, since other forms of expressing relationships may be in place [85].

Valuable relationships may occur among pair partners, direct family members or distantly related kin [86, 97, 98] as well as between unrelated individuals [71, 99] and may involve coalition and alliance formation [100, 101], communal defence [102, 103], communal or cooperative breeding [98, 104–110], conflict resolution [74, 111–115], and social support ([116, 117] and references therein) (see Table 1 for a complete overview). We found support for all these aspects in both altricial and precocial mammals and birds (Table 1). However, whether they occur equally frequently among altricial and precocial species cannot be determined from the available literature.

One notable exception where detailed information on the actual distribution in relation to developmental mode is available is cooperative breeding in birds. Cooperative breeding systems are more common in altricial (11% of 7698 species, including many passerines) than in precocial (4% of 789 species) birds [35, 104, 118]. This is presumably due to the extended need of parental care in altricial nestlings, offering the opportunity for subordinates to increase reproductive success of the breeders through helping ([36, 119], but see [120–123] for examples of cooperative breeding in precocial birds). Although there are several precocial bird species that breed cooperatively, there is a lack of information on their detailed social structure. The only two cases in which we found thorough information, *i.e.* the white-winged trumpeters (*Psophia leucoptera)* and dusky moorhen (*Gallinula tenebrosa*), indicate a polyandrous mating system [122–124]. The male-biased sex ratio in these groups is either due to defence of large permanent territories in order to supply sufficient resources [124], or limited numbers of nest sites [123], which created opportunities for cooperative breeding. In contrast, cooperative breeding in mammals is generally rare (<5% species; [125]) and where it does occur, cooperative breeding appears to be independent of the developmental mode [125]. The classic example is probably found in mole rats (rodent infraorder *Hystricognathi*), which contain solitary, social and a minimum of two eusocial taxa [126]. In the eusocial species, the Damara mole rat (*Fukomys damarensis*) gives birth to precocial young [127], whereas offspring of the naked mole rat (*Heterocephalus glaber)* have been described as altricial [128]. Overall, the independence of developmental mode in cooperatively breeding mammals is presumably due to the fact that precocial offspring in mammals (in contrast to birds) still need substantial parental care (*e.g*. nursing). Thus, extended parental care seems to facilitate cooperative breeding, although the pattern in mammals is less clear than in birds.

Overall, we show that social features are exhibited by both altricial and precocial mammal and bird species. Although differences may exist with regard to cooperative breeding systems, it is likely that this is driven by the greater need for help (*e.g.* feeding offspring) in altricial compared to precocial species, and that this link is unlikely driven by differences in brain size or the capacity for social complexity [129]. It should be mentioned that there is a dispute on whether cooperative breeding should be considered as socially complex, as generally cooperative breeders possess a more stable group composition than fission-fusion societies [130]. This is supported by the idea of Isler & van Schaik [51], who suggest that cooperative breeding in mammals seems independent of advanced cognitive abilities, but that an evolutionary change towards allo-parental care might be a precursor for enlargement of the brain. Still, cooperative breeding requires managing social relationships, although social life may require different skills in various social systems. For cooperative breeders, this includes, for example, the ability to recognise group members, dominance, or kin. Furthermore, even if there might be more complex social systems, cooperative breeding warrants a discussion in this review, as it is the social system with the most detailed information on its distribution in relation to developmental mode.

#### Comparing features of social cognition in precocial and altricial species

Social life may require a need to anticipate, appropriately respond to, cooperate with, or manipulate the behaviour of others. Consequently, behavioural flexibility and some essential cognitive skills are vital (see [131] for a recent review). Accordingly, being part of a complex social environment has frequently been assumed to require high-level cognitive skills and a large brain [4, 15, 18, 43, 132–134], although this relationship may not be as firm as suggested. Larger brains certainly are bigger associative tools with a greater capacity to engage in pattern-recognition and completion, but this does not need to be ‘cognitive’ as usually interpreted. Being longer-lived and more socially complex may require superior pattern-recognition skills as there will be more patterns to be recognised. Accordingly, longer-lived and more socially complex animals will be exposed to larger variability and unpredictability over the course of their life, but this is something different from the kinds of ‘cognitive skills’ that are conventionally given emphasis to and may be achieved not (only) via brain size but also neuronal circuitry. In precocial primates, for example, cooperatively breeding *Callitrichidae* (marmosets and tamarins) outperform their closest relatives, independently breeding squirrel- and capuchin-monkeys (*Cebidae*), in socio- but not non-socio cognitive contexts [135] despite the fact that they possess relatively small brains [136, 137]. Similarly, the lack of a relationship between cooperative breeding and relative brain size in the parvorder *Corvida* [138] argues against such a link. In neither example, however, can we deduce the influence of developmental mode on social cognition as all representatives of the *Corvida* are altricial and all representatives of the *Callitrichidae* are precocial, and comparable data for closely related species that display the opposite developmental mode are not available.

The view that birds are incapable of complex cognitive tasks due to their mainly striatal forebrain has been out-dated since it is now clear that the brain circuits, which regulate social behaviour in non-mammalian vertebrates, are homologous to those found in mammals [22–25]. As the ‘social brain’ hypothesis [4] posits that social complexity and brain size go hand in hand, the developmental mode may, therefore, affect social complexity, particularly in birds. However, the view that complex social interactions indeed require a large brain has recently been challenged [1, 7, 16, 21, 46], which may imply that the relationship between social complexity and developmental mode is less clear as well (see Fig. 1). Social behaviour, which appears cognitively demanding [139], might be achieved through simpler associative mechanisms [21, 140], or probably through a combination of associative learning and more cognitively complex explanations. Furthermore, complex behaviour has been suggested to emerge even from relatively simple nervous systems, and to be the product of not only processes occurring in the brain but of the entire body and the environment [141]. We now will present examples of cognitive abilities we deem crucial for navigating efficiently in a social world, thereby, again, distinguishing between altricial and precocial mammals and birds. Notably, we consider abilities as cognitive irrespective of whether they are presumably simple or complex, following the definition of Shettleworth (pg. 4 [140]) describing cognition as “the mechanisms by which animals acquire, process, store and act on information from the environment ”, which therefore comprises perception, learning, memory, and decision-making.

### Recognizing others

Probably the most vital prerequisite of social complexity is the ability of individuals to recognise others, particularly where multiple individuals with differing intentions interact with one another repeatedly. Such social recognition is an underlying assumption of behaviours including nepotism ([142] for review), several forms of cooperation [143, 144], deception [145, 146] or direct reciprocity [144, 147]. Once again, there is an ongoing debate as to whether the ability to recognise others is cognitively demanding, as it can either be achieved through cognitively simpler means, such as differentiating between more or less familiar individuals (‘class level recognition’ ([148], but see [149]), or through recognition of unique individual features (true individual recognition), which is thought to require specific cognitive adaptations [150]. As both class level recognition or true individual recognition involve cues produced by the signaller as well as perception by the receiver and a specific behavioural response [149], we consider both to require cognitive skills albeit variation in the degree of complexity.

Kin recognition is important for the evolution of social behaviour in many species [151], as it permits indirect benefits of cooperation when individuals improve fitness of relatives [152, 153] or avoidance of kin competition or inbreeding [154–157]. The most commonly studied forms of kin recognition consist of three domains: parent-offspring recognition [158–162], offspring-parent recognition [163–165] and sibling recognition [154, 166–169]. Our survey of the literature indicates that these appear commonly in both precocial and altricial birds and mammals (Table 1). Parent-offspring recognition, based on familiarity or on phenotypic traits, seems to be well developed in cooperative breeders [151, 170–173] presumably because kin-selected benefits often drive the care of others’ offspring [174]. Studies on offspring-parent recognition seem to indicate that being able to identify parents is particularly important in precocial species [26, 163, 175], because there is a higher potential to lose contact with the parent if the offspring is not confined to a nursery, *e.g.* nest or burrow. Sibling recognition has been studied quite intensively and identified in mammals, particularly rodents, and to a lesser extent in both altricial and precocial avian systems ([166, 176], for review [168]). Regardless of developmental mode, some birds and mammals can also recognise unfamiliar kin based on phenotypic traits [155, 156, 177, 178]. Thus, developmental mode in both mammals and birds seems insignificant in the ability to recognise other individuals.

### Long-term memory

Another useful skill of social animals may be the ability to retain information on group members or outcomes of previous interactions over extended periods of time [179]. Although there are not many studies on social memory, those conducted to date seem to indicate that the mode of development has no influence on either the duration that animals may remember socially relevant individuals [180–184] or on keeping track of hierarchical relationships [185–187]. In an extensive literature search, we found only one study that specifically compared learning memory and memory retention (‘recall memory’) in a colour discrimination task in an altricial (domestic Bengalese finch, *Lonchura striata domestica*) and a precocial (blue-breasted quail, *Coturnix chinensis*)) bird [188]. Recall memory is considered to be more cognitively demanding as it is important to remember attributes or relationships between items, relative to recognition memory, where it is enough simply to remember what was seen before. Quails performed poorly in the learning task and failed in the memory retention task, whereas finches scored more highly in the learning task, and retained the ability to discriminate between colours correctly for 45 days, which suggests an influence of developmental mode [188]. However, as this work was performed with domesticated species, it is difficult to determine if this would also hold true for the closest wild relatives. There is only contradictory information on the social system of closely related Japanese quail (*C. japonica*) in the wild [189], but the closest relative of the hybridised Bengalese finch, the white-rumped munia (*L. striata*), is social. The result of the above-mentioned experiment might be a consequence of cognitive skills that are related to sociality, rather than the developmental mode, as, for example, highly social greylag geese (*Anser anser*) memorise dyadic relationships in a hierarchical colour series for approximately one year [187]. Furthermore, there might be other potential causes for differences in learning memory or memory retention in the two species than developmental mode. For such studies to be conclusive they need to provide a higher number of replicate species, which preferably have a more similar biology. This would allow to isolate the effect of developmental mode from other potential effects on the variable studied.

### Remembering and deducing relationships

In social animals, it may pay to not only identify others but also to understand social relationships between other group members, such as who shares a bond with, or who is related to, whom. There are examples of these ‘third party’ relationships in altricial and precocial birds and mammals (Table 1), but the limited number of studies does not allow for claims about whether third party recognition is more prevalent in one developmental mode or the other.

Another advantageous skill would be the ability to deduce the nature of unknown relationships from known ones through indirect evidence, a feature known as transitive inference (TI, [190]). Although transitive inference can be useful in various domains, it is particularly beneficial in the context of social dominance, as it may allow individuals to deduce their dominance relationships with other group members without having to interact with each one of them directly. Once thought as a cognitively-demanding feature of logical thinking and reasoning, it is now recognised that transitive inference can also be achieved through relatively simple associative mechanisms ([191] and [192] for reviews) or probably through a combination of both [193, 194]. Transitive inference has been described across a range of taxa, ranging from fish to primates ([195], Table 2), and although it has been described in altricial birds and mammals as well as precocial birds (Table 1), it has not been tested specifically in any precocial mammal. Therefore, overall, we cannot make firm conclusions about the role of the developmental mode in the ability to deduce unknown relationships. However, the available evidence supports the notion that like for earlier-mentioned features, developmental mode seems to play an ancillary role, but we urge future studies to focus on this phenomenon in a wide range of species.

### Social learning

Numerous studies indicate that individuals pay attention to -and learn from- group members [196–203]. Social learning allows for more appropriate responses to environmental or social cues in various contexts in the future. Both social mammals and birds take advantage of the knowledge of others, irrespective of the developmental mode (Table 1). The lack of a thorough differentiation with respect to developmental modes in the context of social learning tactics, however, does not allow for a decisive evaluation of either the frequencies or variation in altricial and precocial species.

### Other cognitive skills

There are several other cognitive skills in the social domain that may be worth investigating with respect to the developmental modes, (*e.g.* tactical deception [145, 204–208] and other Machiavellian-like behaviours [133, 205, 209]), but we focussed here on the ones we deemed most crucial. As with several of the cognitive skills described above, many of the non-described features have not been studied systematically across the altricial – precocial spectrum, and have been investigated predominantly in ‘large-brained’ mammals and birds. Therefore, at present, we lack the taxonomic breadth to draw sound conclusions about the influence of the developmental mode on any of the cognitive abilities. This clearly also includes some of the features that are listed in this review.

## Conclusion

Our review of the existing literature shows that many mammal and bird species are skilled in a wide range of contexts in the social domain and the existence of these social skills in both altricial and precocial species suggests that social skills are generally irrespective of species’ developmental trajectories. It remains to be investigated if certain aspects of the complexity of various social systems are more common in one developmental mode or the other, and what the evolutionary reason might be. To the best of our knowledge, the only quantitative assessment available is on avian cooperative breeding systems.

There is a need for explicit comparative investigations on variation of the social features in altricial versus precocial species to unravel similarities or differences in, *e.g.* the complexity as well as the quality of bonds displayed in these systems. This includes an evaluation of the different types of affiliative behaviours displayed by altricial versus precocial species to determine whether outwardly different behaviours, in fact, signal, for example, similar strengths of social bonds.

Likewise, both altricial and precocial species are proficient in basic abilities of their social cognition despite established differences in brain size. As this has not been studied systematically, it remains to be determined if this is accomplished via the same underlying mechanisms. That we are in need of integrative studies on sociality, cognition and its accompanying communicative skills in order to decipher how the social environment may form behaviour and brain adaptations for social complexity was recently proposed by Sewall (2015) [131]. We suggest adding to this claim also the indispensable needs to take the developmental trajectories into account. The only study to specifically test cognitive abilities in relation to developmental mode [188] was done in only two species of domesticated birds, which may or may not reflect the natural social environment. Studies in closely related altricial and precocial rodents might be especially suitable for a comparative study in this context.

Altogether, from a qualitative point of view there is little reason to assume that the developmental mode affects social complexity or its underlying cognitive capacities. We do, however, need more quantitative and comparative studies on social complexity in altricial and precocial animals. Yet, as Barrett *et al.* recently stated, “brains evolved as behaviour-control systems designed to help animals move around in, and engage actively with the world” [8]. Indeed, despite the well-established variation in brain size and structure, both altricial and precocial species appear to be able to effectively meander through their complex social world [210].
